# Transcription profiling provides insights into gene pathways involved in horn and scurs development in cattle

**DOI:** 10.1186/1471-2164-11-370

**Published:** 2010-06-11

**Authors:** Maxy Mariasegaram, Antonio Reverter, Wes Barris, Sigrid A Lehnert, Brian Dalrymple, Kishore Prayaga

**Affiliations:** 1 CSIRO Livestock Industries, 306 Carmody Road, St. Lucia 4067, Queensland, Australia; 2 Cooperative Research Centre for Beef Genetic Technologies, Armidale, NSW, 2351 Australia

## Abstract

**Background:**

Two types of horns are evident in cattle - fixed horns attached to the skull and a variation called scurs, which refers to small loosely attached horns. Cattle lacking horns are referred to as polled. Although both the *Poll *and *Scurs *loci have been mapped to BTA1 and 19 respectively, the underlying genetic basis of these phenotypes is unknown, and so far, no candidate genes regulating these developmental processes have been described. This study is the first reported attempt at transcript profiling to identify genes and pathways contributing to horn and scurs development in Brahman cattle, relative to polled counterparts.

**Results:**

Expression patterns in polled, horned and scurs tissues were obtained using the Agilent 44 k bovine array. The most notable feature when comparing transcriptional profiles of developing horn tissues against polled was the down regulation of genes coding for elements of the cadherin junction as well as those involved in epidermal development. We hypothesize this as a key event involved in keratinocyte migration and subsequent horn development. In the polled-scurs comparison, the most prevalent differentially expressed transcripts code for genes involved in extracellular matrix remodelling, which were up regulated in scurs tissues relative to polled.

**Conclusion:**

For this first time we describe networks of genes involved in horn and scurs development. Interestingly, we did not observe differential expression in any of the genes present on the fine mapped region of BTA1 known to contain the *Poll *locus.

## Background

Three genes are believed to control the development of horn phenotypes, namely *Poll*, *African horn *and *Scurs *[1]. The *Poll *gene, which results in the absence of horns, was mapped to the centromeric region of bovine chromosome 1 (BTA1) by Georges *et al*. [2] and has been confirmed by several groups [3-5]. Using 20 microsatellites, the *Poll *locus has been restricted to a 1 Mb interval between the microsatellite markers *RP42-218J17_MS1 *and *BM6438 *in the centromeric region of BTA1 [6]. A report of the location of the *Scurs *locus responsible for producing a loose or "wobbly horn" to BTA19 [7] has not been confirmed [8]. The postulated *African horn *locus has not been mapped to a chromosome [9].

The mapping of the *Poll *locus has not led to the identification of the causative genetic variant for the trait. Mouse genetic models have in the past yielded useful positional candidate genes for important production traits in livestock species, for example *MSTN *(muscle hypertrophy) [10], and *DGAT1 *(milk yield and composition) [11]. As horn development is unique to the *Bovidae*, no small animal models for the phenotype exist, making the identification of candidate genes difficult. Additionally, ambiguities in phenotypic characterisation of horns as distinct from scurs has also precluded the precise mapping and identification of candidate genes for scurs [5,8]. A less constrained approach involves the use of DNA microarrays to uncover transcripts associated with different morphologies, as successfully demonstrated in the identification of calmodulin as a mediator of beak size in Darwin's finches [12]

It is thought that horn development is primarily controlled by the skin [13]. During the initial stages of horn bud development, the epidermis stops making hair and begins to synthesise horn. Once initiated, the primordium of the bony core of the horn forms as a separate centre of ossification in the dermal connective tissue underneath the horn-forming region, which is later fused to the skull [14]. Consequently, gene expression was evaluated in horn bud tissues obtained from the epidermal and dermal tissues of 1-2 week old Brahman calves.

In this study we report results from transcript profiling to identify key genes and molecular mechanisms underpinning the development of horns and scurs in Brahman cattle. We were interested in describing the network of differential gene expression that occurs during the development of the horn and scurs in cattle by contrasting to polled animals. Additionally we wanted to test the hypothesis of a highly differentially expressed gene on BTA1 and 19, for horn and scurs respectively, corresponding to the reported loci.

## Methods

### Sampling

**Figure 1:**
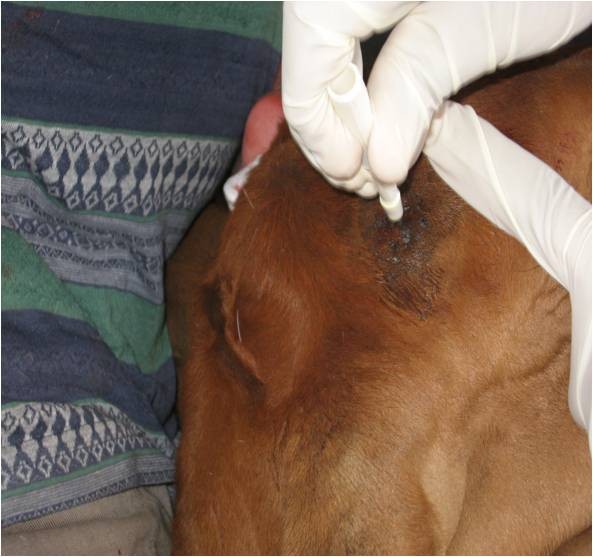
**Tissue sampling from the horn-forming region on the skull using a 3 mm biopsy punch**. The prospective horn-forming region is not apparent always. It was assessed in similar age calves with apparent signs of horn development such as keratinised skin without hair growth or horn bud appearance. Based on these initial observations, the prospective horn region was located in calves with no obvious signs by drawing lines from eye and ear upwards and where they cross each other on both sides of the poll.

All animal experimentation complied with the Animal Ethics requirements of the Commonwealth Scientific and Industrial Research Organisation (approval RH-226/06). Biopsies (average weight 63 mg) that included epidermis, dermis and dermal connective tissue were obtained from the horn-forming region of the skull of prospective horned, scurred and polled Brahman calves using a 3 mm biopsy punch (Paramount Surgimed Ltd., India) as shown in Figure 1. In each case, calves were restrained in a crush and administered with 2 ml Lignocaine 20 at the horn base to numb the skull region. Sampling was carried out at weekly intervals from a few days after birth to about 60 days after birth, although only biopsies from the first and second weeks were used in this study. In each case, tissue biopsies were immediately transferred to cryovials containing RNAlater^® ^solution (Applied Biosystems, Foster City, CA, USA) and placed on ice. The samples were stored in a refrigerator overnight and frozen at -20°C until required. At the time of sampling, the prospective phenotype of the calves was not always apparent, such that the sampled skin area did not differ in appearance from surrounding skin. The horn status phenotypes of the calves were assessed over a period of 1 year to ascertain whether they are polled, horned or scurred.

### RNA extraction, RIN evaluation and hybridization

For RNA preparation, the tissues biopsies were blotted dry to remove excess RNAlater^®^, trimmed of the topmost keratin layer, wrapped in heavy duty aluminium foil and snap-frozen in liquid nitrogen. The frozen samples were physically disrupted by impact and then transferred to 2 ml screw cap tubes containing 1 ml Tri-Reagent (Applied Biosystems) and a mixture of 2- and 4- mm acid washed glass beads. The samples were homogenised in a Thermo-Savant FastPrep^® ^(Thermo Fisher Scientific Inc., MA, USA) for 25 s and the lysate treated as previously described [15]. Total RNA was isolated using the RiboPure™ kit (Applied Biosystems), according to the manufacturer's instructions. All samples that showed OD260/280 ratios > 1.8 were examined on the Agilent 2100 Bioanalyzer (Agilent Technologies, Palo Alto, CA, USA) using an RNA 6000 Nano LabChip (Agilent Technologies) and only those yielding RNA with RNA Integrity Number (RIN) > 6.5 were hybridized to a microarray. From the original samples, one of the horn samples was discarded on account of low RIN resulting in a total of 11 samples (6 male and 5 female) corresponding to the three phenotypic categories of horned (3), polled (4) and scurred (4) from the first week time point. These were hybridized against the Agilent Bovine - Four Plex G2519F 44 k array in a dye swap experiment. Amplification of mRNA, hybridization and data scanning services were performed at the SRC Microarray Facility, University of Queensland http://microarray.imb.uq.edu.au/.

### Agilent array annotation

The bovine Agilent array contains 21,475 unique 60-mer probes, designed to represent approximately 19,500 distinct bovine genes. Probe annotation was accomplished through a series of sequence searching steps using the Baylor College of Medicine Bovine Genome (version 4) as the reference sequence. The probes were aligned to Btau4.0. Both human and bovine reference gene sets were also aligned to Btau4.0. A comparison was then made of the locations of the probes with respect to the locations of the genes. It was noted where a probe overlapped with an intron or an exon of a gene. If there was no overlap, the nearest gene was noted. A direct BLAST [16] of all of the Agilent probe sequences against the most current bovine reference gene set was also performed

The gene prediction for each probe was performed using the following algorithm.

Step 1: If the probe sequence overlapped with a bovine exon and that gene name did not begin with 'LOC or 'MGC' we used that gene name. If there were more than one overlapping bovine gene, the name corresponding to the lowest accession number was used.

Step 2: If the overlapping bovine gene began with either 'LOC' or 'MGC' or when there was no overlapping bovine gene, we used the overlapping human gene name. In the event there was more than one overlapping human gene, the name corresponding to the lowest accession number was used.

Step 3: If there was no overlapping gene, we used the name of the nearest gene based on the alignment to Btau4.0.

Step 4: If there was direct blast hit to the bovine reference set and the name of that gene differed from the one we selected using either of the first two steps, the entry was highlighted to signify uncertainty with the annotation. These annotations were then manually checked to choose the correct gene name or to discard the probe when the issue was difficult to resolve.

### Microarray design and Statistical analysis

**Figure 2:**
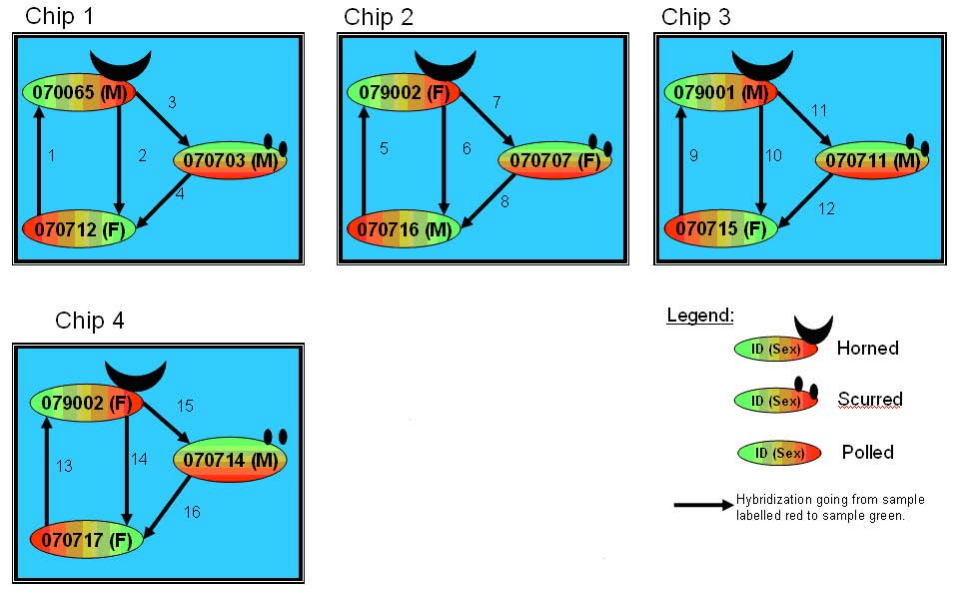
Experimental design

The experimental design had a greater emphasis (i.e. more hybridisations) for the important contrast of horned versus polled (Figure 2). In the four chips, the horned, polled and scurred samples were compared across the 2 sexes (i.e. no confounding between sex and chip) and using RNA samples from independent individuals (i.e. no confounding between individual and chip). Also, care was taken to balance dye channel across samples so that no sample was labelled with the same dye in the two (in case of scurred) or three (in case of polled and horned) hybridisations in which it was used. Alternate dye channels were used to account for systematic effects due to dye bias. The proposed design yielded 8 data sets for each chip (coming from 4 arrays and 2 dyes) distributed across two sexes and three phenotypes.

Gene expression intensity signals were subjected to a series of data acquisition criteria based on signal to noise ratio and mean to median correlation as detailed Tan *et al*. [17]. In brief, we employed the following two editing criteria for data acquisition: First, we required that the signal to noise ratio s > 1, where s is obtained by dividing the background corrected intensity by the standard deviation of the background pixels. Second, we required that the correlation between the mean and the median signal intensities r > 0.85 where r is obtained by dividing the smaller of the mean or median by the larger. Tran *et al*. [18] suggested that a correlation of r ≥ 0.85 not only retains more data than other methods, but the data retained are more accurate than those obtained using traditional thresholds or common spot flagging algorithms. Spots that failed to pass these filtering criteria were assigned a reading of zero. This resulted in a total of 1,375,680 gene expression intensity readings (687,840 of each channel, red and green) that were corrected for background intensity and then log_2 _transformed. The arithmetic mean and standard deviation for the background corrected log transformed red and green intensities were 6.97, SD = 4.21 and 7.90, SD = 3.25, respectively.

Data normalization was carried out using a linear mixed ANOVA model as described in Reverter *et al*. [19] and differentially expressed (DE) genes identified by model-based clustering via mixtures of distributions on the normalized expression of each gene[19,20]. In brief, the following linear mixed model was fitted to the data:(1)

where Y_*ijkhsmn *_represents the *n*-th background-adjusted, normalized base-2 log-intensity from the *g*-th gene (or probe) at the *h*-th phenotype variety (horn status) and *s*-th sex, from the *i*-th chip, *j*-th array (i.e., there are four microarrays per chip) and *k*-th dye channel; μ is the overall mean; C represents a comparison fixed group effects with 32 levels and defined as those intensity measurements from the same chip, array and dye channel; G represent the random gene (or probe) effects with 21,475 levels; AG, DG, HG, SG are the random interaction effects of array by gene, dye by gene, horn-phenotype by gene, and sex by gene, respectively; and finally, ε is the random error term.

In what follows, it is understood that the h-th horn phenotype incorporates the three possible phenotypes. That is: *h = 1, 2*, and *3 *for Polled, Horned, and Scurred, respectively.

For the random effects in Model (1), standard stochastic assumptions are:

where *iid *denotes independently and identically distributed and *N *denotes the normal distribution. Variance components between genes (σ^2^_*g*_), between genes within array (σ^2^_*ag*_), between genes within dye (σ^2^_*dg*_), between genes within horn phenotype (σ^2^_*hg*_), between genes within sex (σ^2^_*sg*_), and within genes (σ^2^_*e*_) were estimated using restricted (to zero error contrasts) maximum likelihood based on the analytical gradients option of VCE6 software ftp://ftp.tzv.fal.de/pub/vce6/.

The solutions for each horn-phenotype by gene interaction from this analysis were of greater interest and were used to generate necessary contrasts to determine which genes were differentially expressed (DE) between two horn phenotypes of interest. The following three t-statistics were computed for each gene in *g*:(2)(3)(4)

Finally, the DE measurement contrasts in (2, 3, 4) were processed by fitting a two-component normal mixture model and posterior probabilities of belonging to the non-null component were used to identify DE genes with an estimated experiment-wise false discovery rate of < 1% as described by McLachlan *et al*. [21].

### Gene ontology distribution and hierarchial clustering analysis

For all of the differentially expressed probes, the corresponding human GeneIDs were analysed in the DAVID database [22] and in Ingenuity Pathway Analysis (IPA, Ingenuity^® ^Systems, http://www.ingenuity.com. This allowed for the identification of enriched biological processes and cellular components specific to the three contrasts of interest, namely, PvH (polled versus horn) and PvS (polled versus scurs). The differentially expressed genes assigned to functional clusters with the DAVID software were also analysed using Permutmatrix [23]. This program performed hierarchial clustering and seriation based on normalised gene expression values to produce a dendrogram. To explore molecular interaction networks among the gene expression profiles, equivalent HUGO gene IDs of the probes were uploaded into IPA Ingenuity Pathway Analysis) software together with the fold changes for each contrast of interest. The program generated networks based on its knowledge base of interactions for the gene list.

### RT-PCR confirmation of DE genes

Validation of the differential expression of genes was carried out for 3 selected genes, namely desmocollin 1 (*DSC1*), desmoglein 1 (*DSG1*) and dehydrogenase 7C (*DHRS7C*), using qPCR. Glyceraldehyde-3-phosphate dehydrogenase (*GAPDH*) was used as the internal reference gene based on the observation of stable expression in animals from all 3 phenotypic classes in the microarray analysis. Taqman gene expression assays for each of these genes were purchased from Applied Biosystems (Assay IDs: Bt03212566_m1, Bt03212583_m1, Bt03241459_m1, Bt03210915_g1 corresponding to *DSC1*, *DSG1*, *DHRS7C *and *GAPDH *respectively).

RNA was isolated from tissue biopsies collected in the second week after birth as described earlier. There were 12 samples in each phenotype category of polled, horned and scurred in the validation experiment with 4 biological replicates and 3 repeats in each sample. All of the animals sampled for the horned phenotype and one of the scurred individual were different from those used for the initial microarray study. In each case, a total of 1 μg of total RNA was treated with Turbo DNA-free kit™ (Applied Biosystems) and 200 ng of the treated RNA was used in cDNA synthesis with random hexamers and the SuperScript™ III First-Strand Synthesis System (Applied Biosystems). The cDNA was diluted one in ten, from which 5 μl was used in subsequent pre-amplification reactions using the Taqman PreAmp Master Mix kit (Applied Biosystems P/N 4384556) according to the manufacturer's instruction. For the Real-time PCR step, amplifications were performed in triplicate on the 7900 Sequence Detection System (Applied Biosystems) using standard cycling parameters.

The relative expression level was determined by subtracting the Ct value (cycle number at the threshold level of log-based fluorescence) of the reference gene from the corresponding target gene across all phenotypic classes of interest. A total of three phenotypic contrasts - polled *vs *horned (PvH), polled *vs *scurs (PvS) and horned *vs *scurs (HvS) - were analysed for statistical significance at each gene. The effects of phenotype, gene and their interaction were included in the generalised linear model with Ct as a dependent variable. The model tested the null hypotheses that Ct differences between target and reference genes were the same between a pair of phenotypes represented as combinational effect (CE): CE1 - CE3 = CE2 - CE4 or CE1 - CE2 - CE3 + CE4 = 0, which yields an estimate of ΔΔCt [24]. For example, in a PvH contrast with *DSC1 *as target gene and *GAPDH *as reference gene, CE1 represents polled-*DSC1*, CE3 represents polled-*GAPDH*, CE2 represents horned-DSC1, and CE4 represents horned-GAPDH. The Proc GLM procedure in SAS (SAS Institute Inc., Cary, NC) was used for the estimation of ΔΔCt and the evaluation of significance.

## Results

### (i) Array annotation results

The results of our annotation process resulted in over 14,000 gene identities assigned to the set of 21,475 probes included on the array (Table 1). This represented a 10 fold improvement in resolution of many of the "LOC" and "MGC" gene predictions in the original annotation from Agilent. The results are not surprising considering the significant strides made in the improvement of the bovine genome assembly since the second version on which Agilent' original annotation was based. However, we still could not determine gene identities for more than 4,000 Agilent probes, based on the algorithm used to check probe sequences against the latest assembly of the bovine genome.

**Table 1:** Results from annotation of probes on the Agilent bovine array

	LOC	MGC	Gene	None
Agilent	12,945	642	3,296	4,681

CLI	995	65	14,904*	898

* This figure does not include 4,613 probes with gene predictions of some uncertainty. CLI refers to the in house annotation.

### (ii) Microarray results

**Figure 3:**
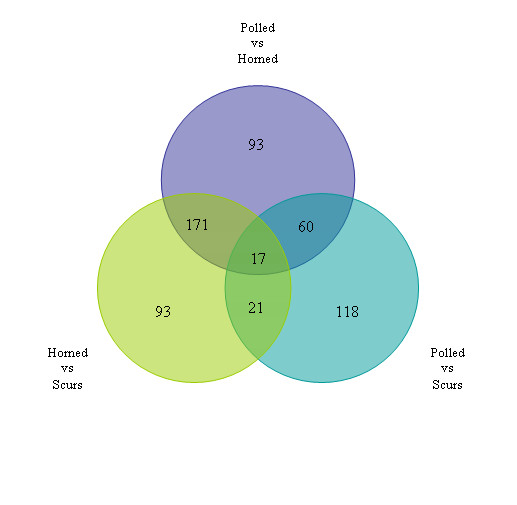
Summary of the 573 differentially expressed genes between the three contrasts of interest i.e. Polled versus Horned (PvH), Polled versus Scurs (PvS) and Horn versus Scurs (HvS)

**Figure 4:**
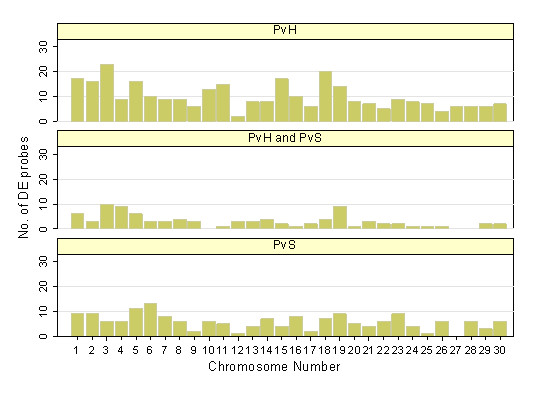
**Distribution of probes on the bovine chromosome (Btau4.0) that were differentially expressed in the Poll Polled versus Horned (PvH) contrast, Polled versus Scurs (PvS) and those that were common to both**. The horizontal axis represents the chromosomal location. Note that number 30 represents the X chromosome.

Approximately 90% of the probes on the Agilent array had signal to noise ratio >1. Of these, a total of 733 probes showed differential gene expression signals between at least one of the three contrasts of interest, namely PvH, PvS and HvS. This converted to 573 genes after considering only those probes that resulted in unambiguous assignment from BLAST and for which ENTREZ gene identities were available (Figure 3). The complete list of probes reporting differential expression is listed in supplementary Table S1. In the PvH contrast, 341 genes were differentially expressed of which 77 were common with the PvS contrast. The highest number of differentially expressed probes in the PvH comparison was observed on chromosomes 3, 5, 18 and 19 with expression changes from -27-fold to 49-fold. For PvS comparison, the highest frequencies of differentially expressed probes were observed on chromosome 5, 6, 19 and 23 (Figure 4) and expression values spanning -14 to 37-fold.

**Figure 5:**
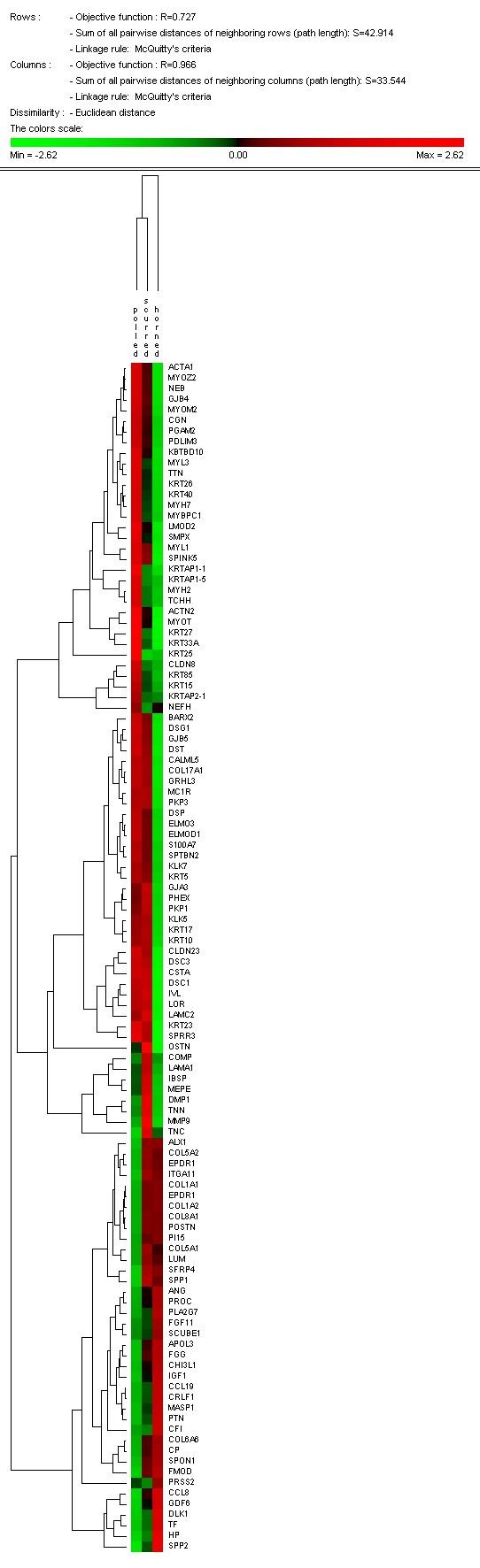
**Genes in overrepresented functional annotation clusters from DAVID analysis were clustered according to their relative gene expression values, using PermutMatrix hierachical clustering software**. Each gene is represented by a single row of coloured boxes; each phenotype is represented by a single column. As shown on the legend, up-regulated genes are indicated with red of increasing intensity and down regulated genes indicated by green of increasing intensity.

For functional clustering analysis purposes, the contrasts of interest were PvH and PvS only. Examination of the differentially expressed genes from the PvH contrast in DAVID (Table 2) identified enriched gene categories corresponding to epidermal development (18 genes), striated muscle contraction (8), intermediate filaments (13), cytoskeleton (41), intercellular junction (14) and extracellular region (28). The most enriched categories in the PvS contrast were for intermediate filament cytoskeleton (11), fibrillar collagen (5) and skeletal development (13). The KEGG Pathways relating to cell communication and extra cellular matrix (ECM) interactions were significantly overrepresented in the PvH and PvS comparisons, respectively. Hierarchical clustering analysis (Figure 5) based on these genes demonstrates expression signatures that set the three phenotypes apart, with scurs intermediary between polled and horned.

**Table 2:** Overrepresented GO terms from functional annotation clustering in DAVID corresponding to those categories of terms with enrichment fold greater than 3.0.

Contrast	Description of change	GO Term	Enriched gene category	Gene number	P-value	Genes
PvH	Genes down regulated in horned versus polled animals	GOTERM_BP_5	epidermis development	18	2.10E-12	COL17A1, KLK5, SPINK5, GJB5, KLK7, IVL, GRHL3, CALML5, KRT5, SPRR3, TCHH, LAMC2, DSP, KRT17, S100A7, LOR, BARX2, KRT10,
	
		GOTERM_BP_5	striated muscle contraction	8	2.40E-07	MYOM2, SMPX, MYBPC1, MYH7, PGAM2, MYH2, KBTBD10, TTN
	
		KEGG_PATHWAY	cell communication	16	2.20E-08	DSC1, KRT40, KRT33A, COL17A1, DSC3, KRT25, GJB5, GJB4, KRT23, KRT5, DSG1, LAMC2, KRT27, KRT17, GJA3, KRT10,
	
		GOTERM_CC_5	intermediate filament	13	6.40E-06	KRT40, KRT33A, KRT25, KRTAP1-1, KRT23, KRTAP1-5, KRT5, KRT26, DSP, KRT27, KRT17, KRT10, PKP1
	
		GOTERM_CC_5	cytoskeleton	41	1.00E-08	ACTN2, KRT40, DSC1, MYL1, PKP3, DSC3, LMOD2, MYH7, NEB, KRTAP1-1, KRT25, CSTA, MYH2, MYOZ2, KRT23, KRTAP1-5, SPRR3, KRT26, MYL3, KRT10, PKP1, KRT33A, ACTA1, KBTBD10, CGN, IVL, ELMOD1, DST, PDLIM3, MC1R, MYOM2, ELMO3, KRT5, MYBPC1, MYOT, SPTBN2, TCHH, KRT27, DSP, KRT17, LOR
	
		GOTERM_CC_5	intercellular junctions	14	1.50E-07	DSC1, COL17A1, PKP3, DSC3, GJB5, CLDN8, CGN, GJB4, DST, CLDN23, DSG1, DSP, GJA3, PKP1
	
	Genes upregulated in horned versus polled animals	GOTERM_CC_ALL	extracellular region	28	4.80E-11	GDF6, IGF1, PROC, ANG, DLK1, PTN, SCUBE1, FMOD, PLA2G7, CHI3L1, SPP2, CCL8, CP, HP, MASP1, SFRP4, SPON1, CFI, COL8A1, APOL3, CRLF1, PRSS2, PI15, FGG, FGF11, CCL19, TF, EPDR1

PvS	Genes down regulated in scurred versus polled animals	GOTERM_CC_5	intermediate filament cytoskeleton	11	7.50E-08	KRT40, KRT33A, KRT15, KRTAP1-5, KRTAP2-1, KRT85, NEFH, KRT26, KRTAP1-1, KRT25, KRT27
	
	Genes upregulated in scurred versus polled animals	GOTERM_CC_5	fibrillar collagen	5	2.40E-07	COL1A2, COL5A2, LUM, COL1A1, COL5A1
	
		GOTERM_BP_5	skeletal development	13	2.80E-08	COMP, PHEX, IBSP, DMP1, OSTN, ALX1, MEPE, COL1A2, SPP1, POSTN, SPP2, COL1A1, MMP9
	
		KEGG_PATHWAY	ECM-receptor interaction	11	1.30E-08	TNC, LAMA1, TNN, COL1A2, COL5A2, SPP1, COL6A6, COL1A1, ITGA11, IBSP, COL5A1

GO term = gene ontology term, BP = biological process, CC = cellular compartment, KEGG = Kyoto encyclopedia of genes and genomes, ECM = extracellular matrix, PvH = polled versus horned and PvS = Polled versus scurs. Down-regulated indicates genes that showing lower expression in one versus the other and up-regulated indicates genes showing higher normalised expression in one versus the other.

**Figure 6:**
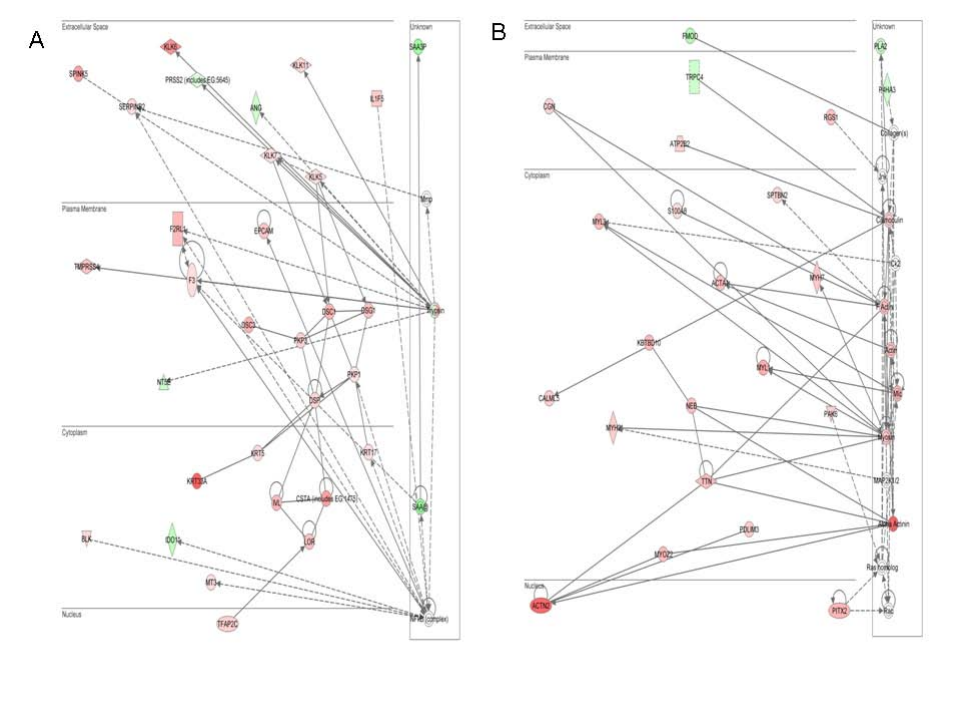
**Top two IPA networks from analysis of all genes differentially expressed in PvH**. The red shading indicates genes that show decreased expression in horned animals versus polled individuals while green is a measure of up-regulation in polled versus horned. The colour intensity is proportional to the level of up- or down-regulation. (A) is the network of genes involved in hair and skin development. (B) Skeletal and muscular system development and function and tissue morphology.

**Figure 7:**
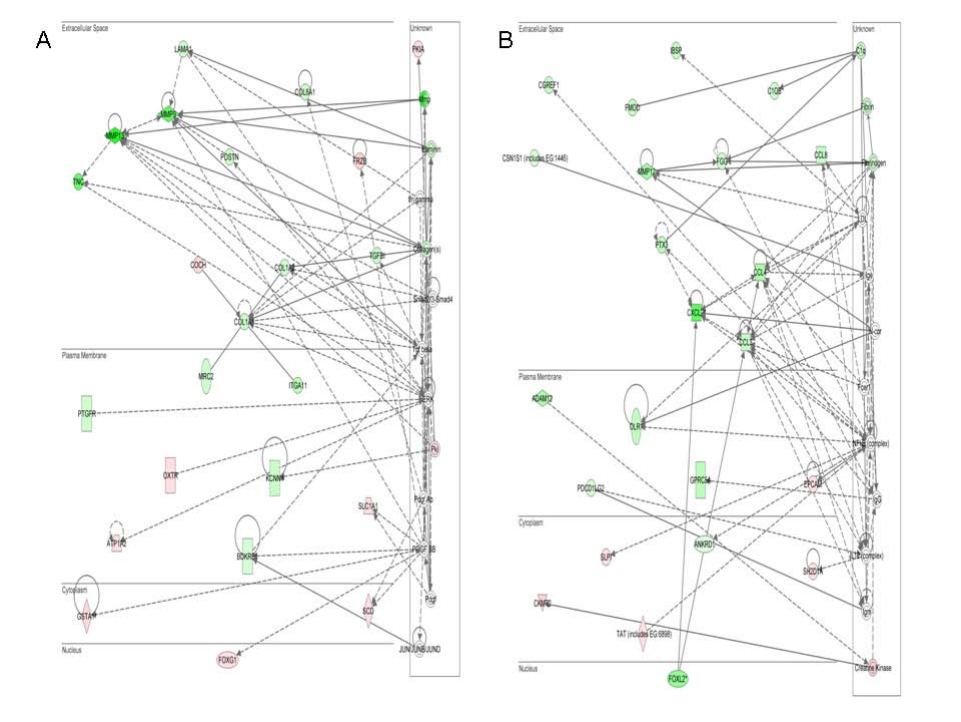
**Top two IPA networks from analysis of all genes differentially expressed in PvS**. Comparisons are with respect to polled animals, consequently the more intense green or red indicate greater up- or down-regulation of the respective genes. (A) Connective tissue development and function/skeletal and muscular system development and function/tissue development. (B) Cellular movement/haematological system development/immune cell trafficking.

Further evaluation of the differentially expressed gene lists in IPA showed enrichment for network pathways involved in hair/skin development and skeletal/muscle development in PvH (Figure 6). The top network pathways in PvS correspond to connective tissue development and immune cell trafficking (Figure 7).

### (iii) Validation of the DE genes by quantitative Real-Time PCR

Gene expression differences detected by the microarray were confirmed with quantitative Real- Time PCR (qPCR) for three genes - *desmocollin *(*DSC1*), *desmoglein *(*DSG*) and *dehydrogenase/reductase (SDR family) member 7C (DHRS7C)- *found to be differentially expressed in the microarray study (Table 3). The selection of *DSC1 *and *DSG1 *was based on their reported functionality in epidermal differentiation while *DHRS7C *was selected based on the observation of differential expression in the microarray HvS contrast and its location within the genome region reported to contain the *Scurs *locus [7]. RNA used for the qPCR validation was isolated from the 2^nd ^week time point after birth and also included animals that were different to those used in the microarray study.

**Table 3:** Changes in expression of DSC1, DSG1 and DHRS7C levels between polled, horned and scurs evaluated by q-PCR.

Contrasts of interest	Gene	ΔΔCt*	**s.e**.	p-value	q-PCR Fold change#	Microarray fold change#
PvH	*DSC1*	-3.48	1.2	0.0075	11.2	8.76
PvH	*DSG1*	-2.95	1.4	0.0402	7.7	7.57
PvH	*DHRS7C*	-7.36	0.6	<0.0001	164	12.6

PvS	*DSC1*	0.99	1	0.3213	-1.98	-0.927
PvS	*DSG1*	0.81	0.9	0.359	-1.87	1.67
PvS	*DHRS7C*	-5.22	1.1	<0.0001	37.3	3.76

HvS	*DSC1*	4.47	1.2	0.0004	-22.2	-9.5
HvS	*DSG1*	3.75	1.2	0.0039	-13.5	-4.56
HvS	*DHRS7C*	2.13	1	0.0459	-4.38	-3.35

*sign of the difference is based on the reference gene corrected 1^st ^phenotype Ct minus the reference gene corrected 2^nd ^phenotype Ct in each contrast for respective target genes.

# fold change is relative to the first phenotype e.g. a change of 11.2 in PvH would indicate that the gene showed 11.2 fold higher expression in polled versus horned tissues

In the microarray analyses, *DSC1 *mRNA showed nearly 9-fold higher expression in polled tissues compared to horned, whereas qPCR found a 11-fold increased expression in polled animals compared to horned individuals. In PvS, the microarray and qPCR showed agreement in level of *DSC1 *expression, where both results were insignificant. *DSG1 *mRNA expression in the microarray study was 7.5 fold compared to 7.7 fold from qPCR in the polled versus horned animals. In PvS comparison, neither microarray nor qPCR revealed changes in *DSG1 *expression levels. *DHRS7C *mRNA expression was 12.6 fold higher in polled versus horned animals compared to 3.8 fold difference between polled and scurred animals from the microarray experiments. The qPCR results show a similar trend of up-regulation of *DHRS7C *gene in polled versus horned and scurs animals, but with much greater fold change.

## Discussion

This study represents the first attempt at transcript profiling to uncover gene expression changes associated with the development of horns and scurs in cattle. Towards this end we sampled epidermal and dermal tissues from the skull region of 1-2 week old calves and analysed the extracted mRNA against the Agilent 44 K bovine array.

The fine mapping of the polled locus to a 1 Mb region on BTA1 [6] and presence of a well characterised 2.5 Mb contig spanning this region [25] have enabled comparative mapping to the human genome, to identify the comprehensive list of genes present in this critical region. Interestingly, none of the genes in the 1 Mb interval encompassing the polled locus were found to be differentially expressed, even though all of them were represented on the microarray. While there were no obvious candidates relating to horn development in this region, it remains uncertain whether the inability to identify differential expression in genes from this region is a consequence of the sampling period. Clearly, if the inductors of horn development preceded the 1^st ^week biopsy or even occurred at the foetal stage, then there would be little chance of detecting their presence in our design. However, a novel regulatory mechanism located on BTA1 igniting these processes involved in cell communication and differentiation cannot be ruled out. In the absence of a clear differentially expressed candidate gene for polled, we analysed the gene list for enriched GO term categories (Table 2) and network relationships between them (Figure 6, Figure 7).

One of the most consistent observations in the differential gene expression results from the PvH comparison was the enrichment for genes relating to skin development as well as striated muscle contraction. Further scrutiny of these genes, indicated that entire modules of genes encoding structural components of cell junctions and attachment show lower levels of expression in horned tissues compared to their age matched, polled counterparts.

Cell junctions are fundamental to cellular attachments and tissue structure, consisting of tight junction made up of cadherins, desmosomes and gap junction proteins [26-28]. The down-regulation of some of these genes or their complexes in horned animals has a striking resemblance to the process of epithelial to mesenchymal transition, also known as EMT. This is both an important developmental and pathological process, whereby the dissociation of the intercellular junction complexes enables epithelial cells to acquire a mesenchymal phenotype [29,30]. In turn, epithelial cells are able to migrate to other environments where they may be involved in tissue morphogenesis [31]. A hallmark of the EMT process involves the loss of intercellular adhesion, in which E-cadherin is vital for its role in promoting homotypic interactions between neighbouring cells [32]. We observed a 4 fold decrease in the expression of *E-cadherin *(*CDH1*) in horned animals compared to polled [Additional file 1].

Another notable feature of the PvH comparison is the lower expression of genes encoding cytokeratin intermediate filaments, principally the keratins and keratin associated proteins in the samples from developing horns, when compared to the samples from prospective polled calves (Table 2). Interestingly many of these keratin genes are present in a cluster on BTA5. Since horns are composed of keratin, at first glance this result does not appear to make sense. Viewed in the context of the EMT, however, the gene expression differences may reflect the remodelling of the epidermis prior to horn formation. Cytokeratins provide the necessary attachment for junction protein to connect to neighbouring cells as well as the extracellular matrix and therefore contributing to the integrity of tissues. In a recent study Liovic *et al*. [33] showed that mutations in keratin genes caused noticeable down-regulation in many of the keratin-interacting components. Consistent with this study, our own observations showed significantly lowered expression of *desomoscollins *(*DSC1*, *DSC3*), *desmogleins *(*DSG1*), *desmoplakins *(*DSP*), *plakophilins *(*PKP1*, *PKP3*), *claudins *(*CLDN 8/11/23*) and *gap junction protein genes *(*GJA3, GJB4 and GJB5*) in horned compared to polled animals, as can be clearly seen in Figure 5 and Figure 6A. The fact that we were able to confirm the differential expression of *DSC1 *and *DSG1 *by q-PCR in 2 week old samples as opposed to week 1 biopsies used in the microarray study suggests that the lowered expression of these adhesion molecules maybe be prolonged for the duration of horn development.

Showing a similar trend of lowered expression in horned compared to polled are two further clusters of genes relating to epidermal differentiation that are present on BTA3 and 18, respectively. The cluster on BTA3 enclodes 4 members of the S100 family of small acidic calcium binding proteins including, loricrin (LOR), small proline rich protein 2A (SPRR2A) and involcurin (IVL). All of these protein products are important constituents of the cornified epithelium. The equivalent human gene cluster, termed "the epidermal differentiation complex" is located on HSA 1q21 [34]. Another cluster on BTA18 codes for the kallikreins, which are serine proteases. Four of the subfamily members (*KLK5*, *KLK6*, *KLK7*, *KLK11*) are down-regulated in PvH. Kallikreins have been implicated in tissue desquamation as well in EMT [35,36]. One could thus envision a "jack pot" effect where an overlapping regulator modulates expression through their interaction with these distinct but functionally related genes.

Beyond the contribution of cytokeratins to the intermediate filament net, the other important structural components of living cells are actin filaments which contribute to cell motility, structure and integrity. Cell migration involves dynamic interactions between the extracellular matrix and the actin cytoskeleton mediated through integrin adhesions. Several intergrin-associated proteins are known to connect the integrins with the actin cytoskeleton such as alpha-actinin and talins among others [37-39]. The expression of *alpha-actinin *is 18-fold lower in horned animals versus polled. An important component of the actin cytoskeleton is myosin II which acts as the bridge that organises actin into higher order structures [40]. Phosphorylation of the myosin II light chain facilitates interaction of the heavy chain with F-actin [41]. Several isoforms of the heavy (e.g. *MYH2*) and regulatory light chains (e.g *MYL1*) of myosin II also show lower expression in horned compared to polled tissues (Figure 6B). Other components of the actomyosin complex that show a similar trend in PvH include *Cingulin *and *Nebulin*. Cingulin has been shown to interact with myosin to link the tight junction proteins to the actomyosin cytoskeleton [42]. Nebulin has been correlated with actin filament length [43]. These changes underscore the flux in cytoskeletal reassembly underpinning horn morphogenesis.

In the PvS comparison, the lower expression of cytokeratin components in scurred animals relative to polled represents the main commonality with the development of horns. However, the main distinguishable feature of scurs was the elevated expression of many members of fibrillar collagen genes which code for components of the proteinaceous extracellular matrix (ECM). For instance both *COL1A1 *and *COL1A2 *show over two-fold increased expression in scurs tissues over polled. Other ECM components showing up-regulation are additional members of the collagen family such as *COL5A1*, *COL5A2 *and *laminin alpha 1 *(*LAMA1*). Some of the most highly expressed genes in the IPA pathways are the matrix metalloproteinases (*MMP9*, *MMP12 *and *MMP13*), *MMP13 *expression is nearly 10-fold higher in scurred individuals compared to polled. The difference in the gene expression of the *MMP *between horned and polled tissues is not statistically significant.

In humans, MMP are represented by 24 genes all of which are collectively capable of cleaving virtually all components of the ECM and the basement membrane [44]. The collagenase activity of MMP [45] supports the induction of the collagens during scurs development. MMP have been shown to have important role during tissue remodelling, wound healing and repair through regulated degradation of the ECM. The other feature of MMP is their involvement in the regulation of a variety of non-matrix substrates such as chemokines, cytokines and growth factors [46] (Figure 7B). When degradation is uncoordinated, MMP have been implicated in arthritis, cancer and cardiovascular disease [47]. A study on laminitis in cattle has also noted the up-regulation of several MMP in ulcerated bovine claw in conjunction with the observation of the disruption of the basement membrane [48].

It remains uncertain whether the lack of greater overlap of differentially expressed genes between PvH and PvS contrasts simply reflects a more advanced state of development in the horn tissue, consistent with the observations that scurs phenotypes in calves takes longer to manifest than horns. The q-PCR validation of greater expression of *DHRS7C *in scurs versus horned tissues is significant in light of its localization on BTA19 and would thus warrant further investigation.

Although there has been no recent study on the physiology of the horn development in cattle, many of the features of horn morphogenesis maybe viewed as equivalent processes to cattle hoof development. The latter has been studied extensively because of the importance of lameness in both cattle and horses. Hoof development involves the intracellular keratinization of epithelial cells and their progressive migration through the suprabasal layers in a program of terminal differentiation, to finally replace the desquamated cornified cells shed from the outermost layers to produce the hardened horn [49].

The down-regulation of cell adhesion molecules that was evident in horn development is consistent with findings reported by Donetti et al. [50]. They found for instance that expression of *Dsc1 *was undetectable in the keratinizing oral mucosa to coincide with keratinocyte terminal differentiation. A similar investigation comparing plakophilin 1 (PKP1) deficient keratinocytes to those expressing recombinant plakophilin 1 found that it had a dramatic effect on desmosomal content [51]. It would thus appear that the breakdown of adhesion and dissolution of the cytoskeleton evident in horn tissues might be setting the stage for keratinocyte transition through the epithelium. Additionally, the enrichment of gene networks relating to EMT may also play a part in the skeletal development of horns. Interestingly, we did not identify changes to cell adhesion molecules in scurs tissues. However, the cytokeratins were down-regulated as in horned tissues. Another feature of scurs was the up-regulation of ECM components which we did not observe in horned tissues.

## Conclusions

In conclusion, this study provides new insights into the dynamics of horns and scurs development. Most of the research into EMT processes has been established *in vitro*, where for instance the actin cytoskeleton and adhesion related molecules have been studied as disparate entities. For the first time we demonstrate all these inter-related molecules working together in a coordinated fashion to produce the horn and scurs phenotypes. Importantly, this work generates new hypothesis on potential regulators of horn development, which linkage studies have mapped to chromosome 1. Although the microarray results indicate a lack of differentially expressed transcript in this region, one cannot rule out a novel regulatory element that was not included in the Agilent array. Recently, Wright et al. [52] showed that a massive duplication of sequences in the intron of *SOX5 *caused the pea-comb phenotype in chicken. Like the horn forming layer in cattle, the pea-comb of chickens is composed of layers of epidermis, dermis and connective tissues. This begs the question whether the polled phenotype is caused by a similar CNV or some structural change on BTA1 that affects entire cascades of genes involved in EMT like processes?

## Authors' contributions

MM carried out the experiment, performed data analysis and drafted the manuscript. AR and KP were involved in the design of the experiment and carried out statistical analysis of the data. BD and WB designed and implemented the algorithm to annotate the Agilent array probes. SL helped with interpretation of the results and drafting of the manuscript. All authors were involved in improving the manuscript.

## Supplementary Material

Additional file 1 **List of all differentially expressed probes The values under "Polled", "Scurs" and "Horned" represent the normalized expression values for each probe/phenotype contrast**. The equivalent human ENTREZ gene identities were processed as described in the methods section. Those genes tagged with an "X" indicate nonconformity to one of the criteria described in the annotation process. These annotations were manually curated to refine the total number of differentially expressed genes to 573 from the total of 733 probes. For each contrast of interest, significance is indicated by "PvH", "PvS" or "PvH". NDE stands for "not differentially expressed". Click here for file
